# Target product profile for readers of rapid diagnostic tests

**DOI:** 10.2471/BLT.23.289728

**Published:** 2023-03-14

**Authors:** Wallace White, Rigveda Kadam, Francis Moussy

**Affiliations:** a FIND, Campus Biotech, Chemin des Mines 9, 1202 Geneva, Switzerland.; b Health Products Policy and Standards Department, World Health Organization, Avenue Appia 20, 1211 Geneva 27, Switzerland.

## Abstract

While equipment-free visual interpretation is a benefit of lateral-flow assays, automating the reading of rapid diagnostic tests promotes proper test performance, interpretation and reporting of the results. We have created a target product profile that describes the minimal and optimal characteristics of various rapid diagnostic test readers. The product profile is intended to promote the development of effective, useful, sustainable rapid diagnostic test readers in support of health programmes worldwide. These readers may be custom hardware or software-only running on a general-purpose mobile device, for professional or lay use, and for medical or non-medical purposes. During the development of the product profile, the World Health Organization and FIND convened a development group of 40 leading scientists, experts, public health officials and regulators. We held a public consultation to which 27 individuals or organizations responded. The product profile calls for rapid diagnostic test readers that at minimum should: (i) interpret colorimetric tests with at least 95% agreement with expert visual interpretation; and (ii) automatically report results and associated data for the health programme. Optimally, readers should: (i) provide at least 98% agreement; (ii) operate with multiple models of rapid diagnostic tests; (iii) assist the user in performing each rapid diagnostic test according to the test instructions; and (iv) offer multiple configurations, operating modes and languages to suit the needs of diverse users, settings and health programmes.

## Résumé

Bien que les tests à flux latéral possèdent l'avantage d'une interprétation visuelle sans nécessiter de matériel, automatiser la lecture des tests de diagnostic rapide favorise la bonne réalisation du test, mais aussi l'interprétation et la communication des résultats. Nous avons donc créé un profil de produit cible qui détaille les caractéristiques minimales et optimales de plusieurs lecteurs de tests de diagnostic rapide. Ce profil de produit vise à promouvoir le développement de lecteurs de tests de diagnostic rapide efficaces, utiles et durables, à même de contribuer aux programmes de santé à travers le monde. Il peut s'agir d'un dispositif logiciel ou matériel sur mesure, fonctionnant sur un appareil mobile ordinaire, à usage professionnel ou non professionnel, et à des fins médicales ou non médicales. Lors de la mise au point du profil de produit, l'Organisation mondiale de la Santé et la Fondation FIND ont réuni un groupe de développement composé de 40 éminents scientifiques, experts, régulateurs et responsables de la santé publique. Nous avons lancé une consultation publique, à laquelle 27 individus ou organisations ont donné suite. D'après le profil de produit, les lecteurs de tests de diagnostic rapide doivent au minimum: (i) interpréter les tests colorimétriques présentant au moins 95% de concordance avec l'interprétation visuelle des experts et (ii) communiquer automatiquement les résultats et les données associées au programme de santé. Idéalement, ces lecteurs doivent: (i) offrir un taux de concordance égal ou supérieur à 98%, (ii) être compatibles avec plusieurs modèles de tests de diagnostic rapide, (iii) aider l'utilisateur à mener chaque test de diagnostic rapide conformément aux instructions et enfin, (iv) proposer de multiples configurations, modes opératoires et langues afin de répondre aux besoins d'un large spectre d'utilisateurs, de lieux et de programmes de santé.

## Resumen

Mientras que la interpretación visual sin equipos es una ventaja de las pruebas inmunocromatográficas de flujo lateral, la automatización de la medición de las pruebas de diagnóstico rápido favorece la correcta realización, interpretación y comunicación de los resultados. Hemos creado un perfil de productos objetivo que describe las características mínimas y óptimas de diversos evaluadores de pruebas de diagnóstico rápido. El objetivo del perfil de producto es promover el desarrollo de evaluadores de pruebas de diagnóstico rápido eficaces, útiles y sostenibles en apoyo de los programas sanitarios de todo el mundo. Estos evaluadores pueden ser de equipo o de programas informáticos personalizados que funcionen en un dispositivo móvil de uso general, para uso profesional o no profesional, y con fines médicos o no médicos. Durante la elaboración del perfil de producto, la Organización Mundial de la Salud y FIND convocaron a un grupo de desarrollo integrado por 40 prestigiosos científicos, expertos, funcionarios de salud pública y reguladores. Celebramos una consulta pública a la que respondieron 27 personas u organizaciones. El perfil de producto exige evaluadores de pruebas de diagnóstico rápido que, como mínimo, deben: (i) interpretar las pruebas colorimétricas con una concordancia de al menos el 95% con la interpretación visual de expertos e (ii) informar automáticamente los resultados y los datos asociados para el programa sanitario. De manera óptima, los evaluadores deberían: (i) proporcionar una concordancia de al menos el 98%, (ii) funcionar con múltiples modelos de pruebas de diagnóstico rápido, (iii) ayudar al usuario a realizar cada prueba de diagnóstico rápido según las instrucciones de la prueba, y (iv) ofrecer múltiples configuraciones, modos de funcionamiento e idiomas para adaptarse a las necesidades de diversos usuarios, entornos y programas sanitarios.

## ملخص

بينما يُعد التفسير البصري، دون اللجوء للمعدات، هو إحدى ميزات فحوصات التدفق الجانبي، فإن أتمتة قراءة اختبارات التشخيص السريع تعزز أداء الاختبار، وتفسير النتائج، والإبلاغ عنها بالشكل الأمثل. لقد قمنا بإنشاء ملف تعريف منتج مستهدف يصف الحد الأدنى والأمثل للخصائص لمختلف أجهزة قراءة اختبارات التشخيص السريع. يهدف ملف تعريف المنتج إلى الارتقاء بتطوير أجهزة لقراءة اختبارات التشخيص تتميز بالفعالية والفائدة والاستدامة، وذلك لدعم البرامج الصحية في جميع أنحاء العالم. قد تكون أجهزة القراءة تلك عبارة عن أجهزة أو برامج فقط مخصصة تعمل على جهاز محمول للأغراض العامة، للاستخدام المهني أو العادي، وللأغراض الطبية أو غير الطبية. أثناء تطوير ملف تعريف المنتج، عقدت منظمة الصحة العالمية وFIND مجموعة تطوير تضم 40 من كبار العلماء، والخبراء، ومسؤولي الصحة العامة، وواضعي اللوائح. قمنا بإجراء استشارة شعبية استجاب لها 27 فردًا أو منظمة. يستهدف ملف تعريف المنتج أجهزة قراءة لاختبارات التشخيص السريع التي يجب على الأقل أن تقوم بما يلي: (1) تفسير الاختبارات اللونية بنسبة اتفاق %95 على الأقل مع التفسير البصري الخبير، و(2) الإبلاغ تلقائيًا عن النتائج والبيانات المرتبطة ببرنامج الصحة. على النحو الأمثل، يجب على أجهزة القراءة أن تقوم بما يلي: (1) تقديم موافقة بنسبة %98 على الأقل، و(2) العمل باستخدام نماذج متعددة من اختبارات التشخيص السريع، و(3) مساعدة المستخدم في إجراء كل اختبار تشخيصي سريع وفقًا لتعليمات الاختبار، و(4) تقديم تكوينات، وأوضاع تشغيل، ولغات متعددة لتناسب احتياجات المستخدمين، والإعدادات، والبرامج الصحية المتنوعة.

## 摘要

虽然无需设备的可视化解读是侧流分析的一个优势，但快速诊断测试的自动化读取有利于提高测试性能、以及检测结果解读和报告的准确性。我们创建了一个目标产品简介，描述了各种快速诊断测试读取器的最基本和最佳特性。该产品简介旨在促进开发有效、有用、可持续的快速诊断测试读取器，以支持全球的健康计划。这些读取器可以是定制硬件或仅在通用移动设备上运行的软件，用于专业或非专业用途，以及用于医疗或非医疗目的。在产品简介的开发过程中，世界卫生组织和全球创新诊断基金会 (FIND) 成立了一个由 40 名顶尖科学家、专家、公共卫生官员和监管人员组成的研发小组。我们举行了一次公众咨询，有 27 个个人或组织做出了回应。产品简介要求快速诊断测试读取器应至少具备以下条件：(i) 在对比色测试结果进行解读时，与专家的视觉解读至少有 95% 的一致性以及 (ii) 自动报告结果和健康计划的相关数据最理想的情况，读取器应具备以下条件：(i) 提供至少 98% 的一致性，(ii) 可操作多种快速诊断测试模式，(iii) 可以协助用户根据测试说明进行每项快速诊断测试，以及 (iv) 提供多种配置、操作模式和语言选项，以满足不同用户、环境和健康计划的需要。

## Резюме

Хотя визуальная интерпретация без использования оборудования является преимуществом иммунохроматографического анализа, автоматизация считывания результатов экспресс-диагностики способствует надлежащему выполнению теста, интерпретации и представлению результатов. В результате был разработан целевой профиль продукта, который описывает минимальные и оптимальные характеристики различных устройств для считывания результатов диагностических экспресс-тестов. Профиль продукта предназначен для содействия разработке эффективных, практичных, надежных устройств для считывания результатов диагностических экспресс-тестов в поддержку программ здравоохранения во всем мире. Эти устройства для считывания могут представлять собой специальное оборудование или программное обеспечение, работающее на мобильном устройстве общего назначения для профессионального или обычного использования, а также для медицинских или немедицинских целей. В ходе разработки профиля продукта Всемирной организацией здравоохранения и Фондом для инновационных и новых диагностических средств (Foundation for Innovative New Diagnostics, FIND) была созвана группа разработчиков в составе 40 ведущих ученых, экспертов, представителей органов здравоохранения и регулирующих органов. Была проведена консультация с общественностью, в которой приняли участие 27 человек или организаций. Профиль продукта предполагает наличие устройств для считывания результатов диагностических экспресс-тестов, с помощью которых по меньшей мере можно: (i) интерпретировать результаты колориметрических тестов с не менее чем 95%-м согласием с визуальной интерпретацией эксперта и (ii) автоматически сообщать результаты и связанные с ними данные для программы здравоохранения. В оптимальном случае считывающие устройства должны: (i) обеспечивать согласованность результатов на уровне не менее 98%, (ii) работать с несколькими моделями диагностических тестов, (iii) помогать пользователю в проведении каждого диагностического теста в соответствии с инструкцией к тесту и (iv) предусматривать несколько конфигураций, режимов работы и языков для удовлетворения потребностей различных пользователей, условий и программ здравоохранения.

## Introduction

Lateral-flow rapid diagnostic tests continue to play a vital role in global health in the management and diagnosis of infectious diseases, including malaria, human immunodeficiency virus and coronavirus disease 2019 (COVID-19). Visually interpreted rapid diagnostic tests, more than any other class of diagnostics, fulfil the World Health Organization’s (WHO) ASSURED criteria (Affordable, Sensitive, Specific, User-friendly, Rapid and robust, Equipment-free and Deliverable to end-users),^1^ enabling their use at the lowest levels of health care and in self-testing.^2^ Their utility is, however, compromised every time a test is incorrectly performed or interpreted or its result is not available in a timely manner for clinical decision-making and surveillance. As companion tools, rapid diagnostic test readers promote more consistent, accurate test performance, interpretation and reporting, as recognized in a revision of the ASSURED criteria^3^ and in comparisons of manual and automatic reports of positivity.^4^

This target product profile addresses various types of rapid diagnostic test readers, with no prioritization: (i) a dedicated hardware instrument or an application that operates on a general-purpose mobile device such as a tablet or phone; (ii) a test reader designed for professional use by a health-care worker or other representative of a health programme (such as a disease control programme, a laboratory service of a health ministry or a private health-care system) or for lay use (i.e. self- and home testing); (iii) one that acts within the narrow bounds of a non-medical reader, recording the user’s interpretation of the test as the definitive result and transmitting the reader’s interpretation only for non-medical uses such as public health surveillance, monitoring, evaluation and external quality assessment; or one that serves as a medical reader, which provides its interpretation to its user as a basis for diagnosis and treatment as a regulated medical device or in vitro diagnostic; and (iv) a test reader offered by the manufacturer of the rapid diagnostic test or provided independently for use with one or more rapid diagnostic test brands.

Where a statement in this target product profile applies only to one type of reader, it is preceded by the name of that type, as summarized in Box 1.

Box 1 **Types of rapid diagnostic test readers in the target product profile**

***Device architecture***

Instrument: dedicated hardware, as most readers have been.

Application: software that operates on general-purpose mobile devices.

***User***

Professional use: by a health-care worker.

Lay use: self-testing and similar use by the general public.

***Intended use***

Medical: shows its interpretation of the rapid diagnostic test to the user.

Non-medical: does not show its interpretation to the user.

Both send data to the health programme.

***Optical technology***

Colorimetric: detection from changed presence, intensity or colour of a line illuminated by visible light; includes tests that can be read visually, with no instrument.

Fluorescent: detectable, typically in otherwise darkness, by emission of a line near one wavelength when excited near another wavelength.

Luminescent: similar to fluorescent but without excitation.

Most readers in the category of device architecture are of one type or the other, but a reader may have aspects of each, for example, an application that requires a physical accessory to hold the rapid diagnostic test in front of the camera, or an instrument that relies on an application on a wirelessly connected phone. Such hybrid readers have certain application-only and other instrument-only characteristics.

While all readers must be capable of reporting results automatically to the associated health programme, the complete set of data features specified by this target product profile need not be available in the reader itself if those features are available in a digital health system with which the reader is integrated. Features for the management and analysis of the data generated by readers are not covered in this target product profile.

This target product profile is a companion to diagnostic target product profiles that state the required characteristics of a particular test. Some aspects of a reader, such as diagnostic sensitivity and specificity, can be evaluated only as a system of a particular test plus reader and should follow the requirements in the relevant diagnostic target product profile.

For each characteristic of the target product profile, an optimal criterion is to be achieved by product developers if feasible and a minimal criterion if the optimal is not feasible. In other cases, the optimal and minimal criteria are the same

## Methods

This target product profile was developed according to a process based on the WHO *Target product profiles, preferred product characteristics, and target regimen profiles: standard procedure, version 1.03*, dated 7 December 2021.

The process was led by Francis Moussy at WHO and Wallace White and Rigveda Kadam at FIND, with advice from Sarah Charnaud at WHO.

The need for a target product profile was assessed in September 2021 and confirmed within WHO. After a review of previous target product profiles, diagnostic practices and need, an initial draft was written. The lead authors created a target product profile development group of 40 leading scientists, experts, public health officials and regulators in this field, with due attention to geographical and gender representation. All members completed the WHO declaration of interests form, with the provision that feedback from members with declared interests in this field would be analysed separately (Table 1 and Table 2).

**Table 1:** Members of the target product profile development group with no conflict of interest, by affiliation at the time of participation

Name	Affiliation(s)	Country of residence
John Bimba	Bingham University Nigeria; Zankli Research Centre	Nigeria
Mary Garcia	Royal Melbourne Hospital	Australia
Mohammed Majam	Ezintsha, University of the Witwatersrand	South Africa
Mothepane Phatsoane Gaven	Ezintsha, University of the Witwatersrand	South Africa
Lara Noble	Wits Diagnostic Innovation Hub, University of the Witwatersrand	South Africa
Lesley Scott	Wits Diagnostic Innovation Hub, University of the Witwatersrand	South Africa
Muzamil Mahdi Abdel Hamid	Institute of Endemic Diseases, Medical Campus, University of Khartoum	Sudan
Jane Akinyi Aduda	Jomo Kenyatta University of Agriculture and Technology	Kenya
Rosanna Peeling	London School of Hygiene & Tropical Medicine	United Kingdom
Cédric Yansouni	McGill University Health Centre, Divisions of Infectious Diseases and Medical Microbiology; J.D. MacLean Centre for Tropical Diseases, McGill University	Canada
Nikki Pai	McGill University; Research Institute of the McGill University Health Centre	Canada
Abidan Nambajimana	University of Gitwe; Partners in Health; Stansile	Rwanda
Kaiser Shen	United States Agency for International Development	USA
Ngor Pengby	National Centre for Parasitology, Entomology and Malaria Control	Cambodia
Michael Aidoo	Centers for Disease Control and Prevention	USA
Valter Pereira de Oliveira	Health Regulatory Agency	Brazil
Paulyne Wairimu	Pharmacy and Poisons Board; African Union Development Agency–New Partnership for Africa’s Development	Kenya
Trevor Peter	Clinton Health Access Initiative	USA
Najma A. Salim	Clinton Health Access Initiative	Kenya
Azraa Mohamed	Clinton Health Access Initiative	South Africa
Purnima Ranawat	Catalyst Management Services/ Swasti	India
Andualem Aklilu	KNCV Tuberculosis Foundation	Ethiopia
Yohannes Demissie Babo	KNCV Tuberculosis Foundation	Ethiopia
Stijn Deborggraeve	Médecins Sans Frontières	Belgium
Amber Sheets	Population Services International	Zimbabwe
Bernhard Weigl	Global Health Laboratories; University of Washington	USA
Denise Habimana	PATH	USA
Shiri Brodsky	PATH	USA

**Table 2:** Members of the target product profile development group with a conflict of interest related to their roles with companies, whether for- or non-profit, relevant to rapid diagnostic test readers, by affiliation at the time of participation

Name	Affiliation(s)	Country of residence
Clayton Sims	Dimagi	USA
Peter Lubell-Doughtie	Ona	USA
Bryan Richards	SystemOne	South Africa
Emily Adams	Mologic; Liverpool School of Tropical Medicine	United Kingdom
Paul Isabelli	Audere	USA
Thomas Scherr	Pragma Health; Vanderbilt University	USA
Ling Koh	Becton Dickinson	USA
David Bermejo Peláez	Spotlab	Spain
Jack Richards	ZiP Diagnostics; Royal Melbourne Hospital	Australia
Patrick Vaughan	DCN Diagnostics	USA
Patrick Coffey	DCN Diagnostics	USA
Santiago Ferro	Ferro Consulting; Fio	Canada

**Fig. 1:**
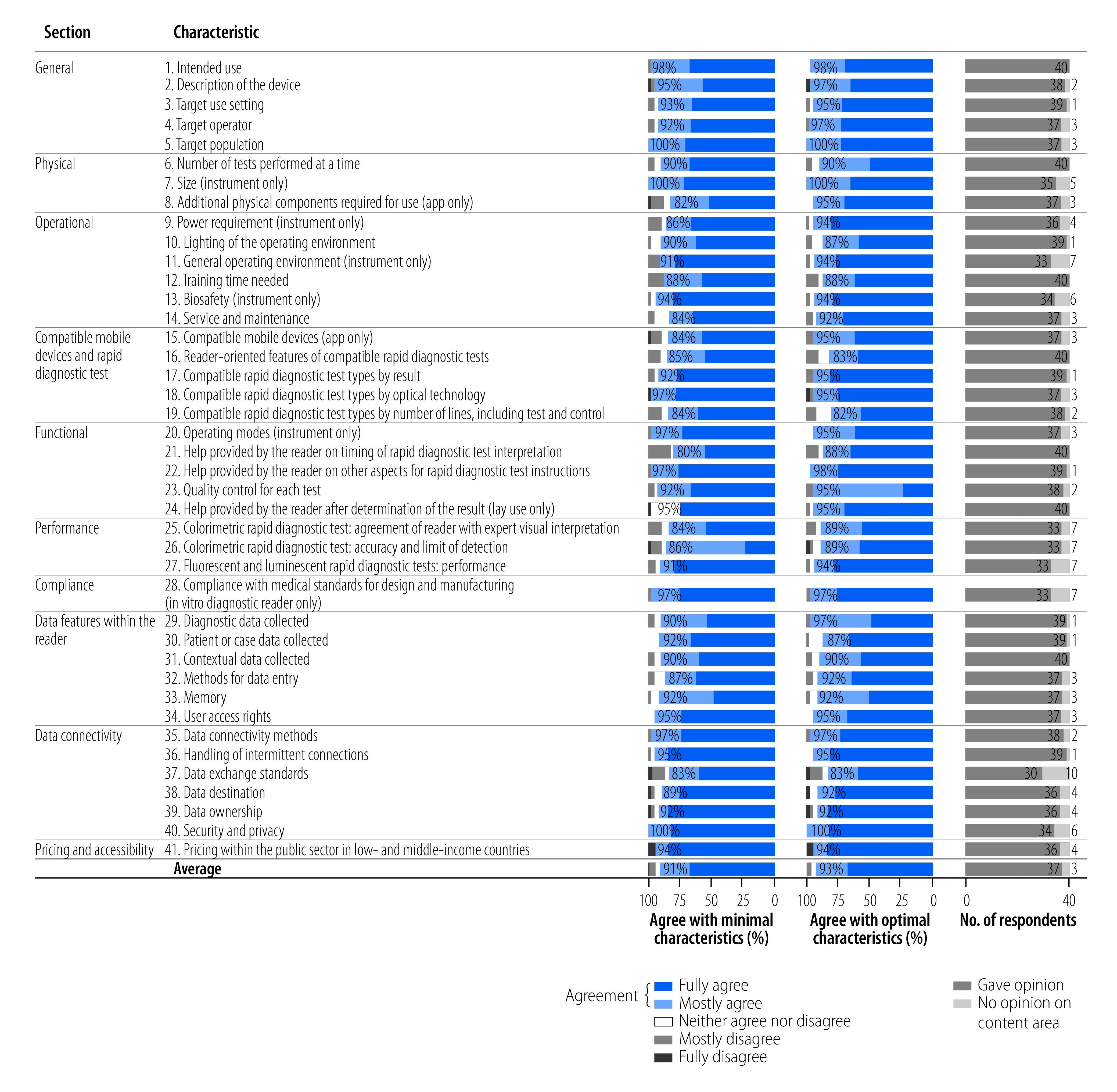
Distribution of Likert scores in the survey completed by the target product profile development group for rapid diagnostic test readers

Note: Adapted from World Health Organization.^5^

During February and March 2022, all development group members received a draft of the target product profile and completed an online survey to elicit their scores for every minimal and optimal item in the target product profile of their rating of agreement on a Likert scale: 1, fully disagree; 2, mostly disagree; 3, neither agree nor disagree; 4, mostly agree; and 5, fully agree. As an alternative to a Likert score, members could mark “No opinion on content area.” Comments were requested on all items and were required when members marked that they did not agree (Likert score, 1–3). The levels of agreement (considered as “mostly agree” or “fully agree”) were high, averaging 92% and at least 80% for every item, as shown in Fig. 1. The lead authors reviewed all comments regardless of Likert score and revised the target product profile accordingly, when practical, to address a criticism, incorporate a suggestion or avoid a clear misunderstanding of the intent.

Once the lead authors had agreed on the next draft, WHO posted it, with announcements on mailing lists and social media by WHO and FIND teams, for a public consultation over 28 days during August and September 2022. Anyone could respond by completing a typical WHO submission form, identifying themselves and listing their comments and suggested amendments.

A total of 27 individuals or organizations submitted 137 requests for changes. In the same way as for the survey of the development group, the lead authors reviewed all comments and revised the target product profile when appropriate and feasible. The proposed revisions to the performance section were reviewed by the development group members without conflicts of interest (Table 1).

WHO then released the current version of the target product profile.^5^

## Profile

Below information is available in a tabular format in the published target product profile.^5^

### General

#### 1. Intended use

##### Minimal

Non-medical: To collect user interpretations and other data from rapid diagnostic tests. 

Medical: To interpret rapid diagnostic tests to aid clinical decisions and to collect other data from rapid diagnostic tests.

##### Optimal

Same as minimal, plus: To support proper test performance.

##### Minimal and optimal

The reader may be used in screening, diagnosis or management of disease. The reader transmits test data, patient data entered by the user and contextual data to the health programme. 

##### Notes

A non-medical reader does not show its interpretation to the user. For medical readers, in most countries each combination of reader and test is subject to medical regulation.

#### 2. Target use setting

##### Minimal and optimal

All levels of health care as well as non-health-care settings.^6^

##### Notes

Examples of non-health-care settings: homes, schools, workplaces, transport hubs, high-traffic areas.

#### 3. Target operator

##### Minimal and optimal

Professional use: Health-care worker, including community health worker, with at least basic literacy and minimal training or any health-care worker with additional training. 

Lay use: Person with at least basic literacy but no formal education in a relevant field of health care or medical discipline. 

Application: Person with access to a mobile device and basic application skills. 

The operator may have requirements for access that must be met by the reader.

##### Notes 

Examples of lay users: self-testers, caretakers, workplace screeners, school screeners. Accessibility: To design a reader appropriately, see *Web content accessibility guidelines*^7^ and characteristic 28 (that is, patient or case data collected).

### Physical

#### 4. Number of tests performed at one time

##### Minimal 

Instrument and application: Operate with one test at a time.

##### Optimal 

Instrument: Offered in two or more configurations: single-bay, operates with one test at a time; and multi-bay, operates with multiple tests in parallel, with random access and capability of testing different analytes simultaneously.

Application: Interprets one test at a time, and can guide administration of multiple tests in parallel, tracking the timing of each. 

##### Notes 

See characteristics 20 (that is, help provided by the reader in timing of rapid diagnostic test interpretation) and 21 (that is, help provided by the reader on other aspects of rapid diagnostic test instructions) on timing of interpretation. Parallel, random-access testing: In “walk away” mode, while one test develops in the reader, a user can start additional tests for more analytes for the same patient or for other patients. A multibay instrument may therefore provide throughput of multiple single-bay instruments at lower cost, space or administration, although with less flexibility and more complexity for the user

#### 5. Size (instrument only)

##### Minimal and optimal 

Small, portable table-top device.

##### Notes 

Appropriate size may depend on the features of the instrument. The instrument should be designed to be moved easily and to withstand drops and impacts associated with portable (not necessarily hand-held) devices (see characteristic 28, that is, patient or case data collected).

#### 6. Additional physical components required for use (application only)

##### Minimal 

Acceptable if they are readily portable and nearly universal (not specific to a few models of mobile devices or rapid diagnostic tests).

##### Optimal 

None.

##### Notes 

Acceptable example for minimal: an optical calibration card. Dependence on such components is not optimal because of costs, logistics and (if not single-use) maintenance.

### Operational

#### 7. Power requirement (instrument only)

##### Minimal

Local 100–240 V AC, 50 or 60 Hz mains power.

##### Optimal 

Same as minimal, plus: User-replaceable rechargeable battery sufficient for an 8-hour shift or user-replaceable single-use batteries.

##### Notes 

The reader’s documentation should explain the electrical interfaces, including power consumption, cord length, mains plug style and single-use battery model, so that implementers can plan accordingly.

#### 8. Lighting of the operating environment

##### Minimal 

Any setting in which the user can see well enough to run the test. Infrequently, the reader may signal that it cannot operate in the current lighting and should indicate how the lighting should be changed to enable operation.

##### Optimal 

Any setting in which the user can see well enough to run the test.

##### Notes 

Examples: indoors without artificial lighting; indoors with no windows and fluorescent lighting; mixed lighting; outdoors in direct sunlight; outdoors in dappled, moving shadows from a tree; or outdoors in shade with indirect sunlight bounced off a red or blue wall.

#### 9. General operating environment (instrument only)

##### Minimal 

10–40 °C and ≤ 90% non-condensing humidity at an altitude ≤ 2500 m; can withstand dusty conditions and water splashes.

##### Optimal 

5–45 °C and ≤ 98% non-condensing humidity at an altitude ≤ 4000 m; can withstand dusty conditions and water splashes.

#### 10. Training for operation

**Minimal**

Professional use: ≤ 2 hours with options for remote or self-training.

Lay use: No training necessary. The user must be able to use the reader correctly when presented with it, its instructions for use and any other labelling.

##### Optimal 

Professional use: ≤ 1 hour with options for remote and self-training. Support provided for training of trainers.

Lay use: same as minimal, plus: The reader enables frequent users to perform tests in an abbreviated workflow that is appropriate for them. (New users are expected to require more support.)

##### Notes 

Professional use: Assumed that users already have experience with rapid diagnostic tests. In view of the roles of professional users and the features of professional readers, these products typically do require training.

Lay use: Assumed that users lack experience with rapid diagnostic tests and that the rapid diagnostic test is designed for lay use. Like self-test rapid diagnostic tests, a self-test reader must be designed and demonstrated to be usable without training. 

For both professional and lay use, see also characteristics 21 (that is, help provided by the reader on other aspects of rapid diagnostic test instructions) and 22 (that is, quality control for each test).

#### 11. Biosafety (instrument only)

##### Minimal and optimal 

Easy decontamination of surfaces with 70% isopropyl alcohol, 70% ethyl alcohol or a bleach solution with 0.5% chlorine.

#### 12. Service and maintenance

##### Minimal 

Instrument: Weekly maintenance (including any software updates) of < 10 minutes by an operator; mean time to failure of ≥ 36 months or 30 000 tests; self-check alerts operator to instrument errors or warnings; operator-involved calibration check at set intervals.

##### Optimal 

Instrument: No maintenance required; software updated automatically or manually, depending on health programme preference; mean time to failure of ≥ 48 months or 40 000 tests; self-check alerts operator to instrument errors or warnings; no operator-involved calibration check required. 

##### Minimal and optimal 

Application: Software updated automatically or manually depending on health programme preference and within the limits of the update policy of each mobile device, as necessary to ensure compatibility with the latest operating system.

### Compatible mobile devices and rapid diagnostic tests

#### 13. Compatible mobile devices (application only)

##### Minimal 

The application maker shall publish and maintain a list of Android mobile devices and operating system versions, including low-priced devices and older versions, that have been determined to be compatible with the application. Devices that use the application shall remain functional for other applications and uses.

##### Optimal 

Same as minimal, plus: Most Android (lay use: and iOS and iPadOS) mobile devices with a rear-facing camera that are readily available in low- and middle-income countries. The application maker may provide an optical performance check to allow users to enable operation on any device that passes the check.

##### Notes 

At present, the capability of the camera of a mobile device for rapid diagnostic test analysis, particularly as a medical reader, is difficult if not impossible to determine from its advertised specifications. The minimal may be adequate for health programmes that provide mobile devices to their health-care workers. The optimal is intended for use in scenarios with less control, including bring-your-own device.

#### 14. Reader-oriented features of compatible rapid diagnostic tests

##### Minimal 

The reader’s manufacturer shall publish and maintain a list of compatible rapid diagnostic test models. The reader shall be compatible with rapid diagnostic tests that have user markings on them.

##### Optimal 

Same as minimal, plus: The reader shall be compatible with rapid diagnostic tests that were not necessarily designed for use with a reader, e.g. rapid diagnostic tests with no computer-vision-friendly markings; strips without a plastic cassette (dipsticks). The reader shall be compatible with multiple brands and types of rapid diagnostic tests. The reader shall use one-dimensional and two-dimensional barcodes, if present on the rapid diagnostic tests, to identify the model, lot, expiration or serial number.

##### Notes 

User markings: It is common for users to write the name of the patient or another identifier on the rapid diagnostic test. 

No medical reader is expected to be universal in the sense of reading all rapid diagnostic test models, as the reader’s performance must be validated with each rapid diagnostic test model. 

Barcoded serial numbers can enable tracing of each test, verification of authenticity and prevention of reuse, but they may remain rare in low- and middle-income countries, partly because of the cost. In view of lack of standardization of their content, the design of barcodes should be integrated between reader and rapid diagnostic test, which is beyond the scope of this document.

#### 15. Compatible rapid diagnostic test types by result type

##### Minimal 

Qualitative.

##### Optimal 

Qualitative; semiquantitative threshold, by comparison of intensity of a test line to a reference; semiquantitative levels, such as low, medium or high; and quantitative.

##### Notes 

The design of semiquantitative and quantitative tests may require integration between reader and rapid diagnostic test, which is beyond the scope of this document.

#### 16. Compatible rapid diagnostic test types by optical technology

##### Minimal 

Instrument: Colorimetric (visible), fluorescent or luminescent lateral flow assays.

##### Optimal 

Instrument: Colorimetric (visible), fluorescent and luminescent lateral flow assays.

##### Minimal & Optimal 

Application: Colorimetric (visible) lateral flow assays.

##### Notes 

The design of fluorescent and luminescent tests may require integration between the reader and rapid diagnostic test, which is beyond the scope of this document. 

#### 17. Compatible rapid diagnostic test types by number of lines, including test and control

##### Minimal 

2 or 3 lines.

##### Optimal 

2, 3, 4 or more lines.

##### Notes 

Example of a three-line test: a malaria test with a control line and lines for *Plasmodium falciparum* and *P. vivax.*

### Functional

#### 18. Language support

##### Minimal 

For each country in which the reader is deployed, one popular language, such as the official language or de facto national language, and any language mandated by local regulatory or trade compliance requirements.

##### Optimal 

Same as minimal, plus: Additional languages to enable use by other residents of that country.

#### 19. Operating modes (instrument only)

**Minimal**

The reader provides a readnow mode, in which the user presents the test to the reader and the reader promptly interprets the test.

##### Optimal 

The reader provides a read now mode (see minimal) and a walk away mode, in which the user presents the test to the reader at the start of the development period, and the reader controls when the test is interpreted.

##### Notes 

In a walk away mode, the reader may be able to release a positive result before the development period has elapsed, if the rapid diagnostic test manufacturer provides for this.

#### 20. Help provided by the reader in timing of rapid diagnostic test interpretation

##### Minimal 

None.

##### Optimal 

In a read now mode, the reader shall provide a countdown timer to prompt the user when the rapid diagnostic test is ready to be read and before expiration of the reading period. The reader shall set the times according to each rapid diagnostic test’s quick reference instructions or job aid. The possibility of overriding the elapsed time limits shall be configurable by the health programme. 

Application: During the countdown, the user shall be able to use the mobile device for other tasks and still receive a prompt from the application when the rapid diagnostic test is ready to be read.

##### Notes 

Regarding elapsed time data, see characteristics 23 (that is, help provided by the reader after determination of the result; lay use only) and 29 (that is, contextual data collected).

#### 21. Help provided by the reader on other aspects of rapid diagnostic test instructions

##### Minimal 

None.

##### Optimal 

The reader shall provide the user with access to rapid diagnostic test instructions equivalent to the quick reference instructions or job aid. The reader may provide enhanced instructions, such as videos, audio and photographic examples of results.

##### Notes 

Minimal: Users should follow regular instructions for use of the rapid diagnostic test, as they would without the reader. 

Optimal: If the reader provides enhanced instructions, their role in regulatory authorization of the test should be considered, as should their usability for both new and frequent users (see characteristic 12, that is service and maintenance). 

Regardless of this characteristic, the reader will include instructions for use, such as how to place the rapid diagnostic test and properly illuminate it.

#### 22. Quality control for each test

##### Minimal 

Check of the rapid diagnostic test’s control line. 

Instrument: Check of the optical system. 

Application: Check of sufficient photographic quality. 

Check of sufficient clearance of the sample. 

Failures will result in warnings to the user and in the record, and critical failures will prevent release of a result.

##### Optimal 

Same as minimal, plus: Check of elapsed time to reading of result; check of sample applied to wrong well; check of expiration by date. Professional use: Check of expected results when running quality control samples.

#### 23. Help provided by the reader after determination of the result (lay use only)

##### Minimal 

The reader displays basic result terms such as positive, negative and invalid.

##### Optimal 

The reader displays extended result messages from the quick reference instructions of each test. Each health programme can provide messages for each type of result of each test, with referral and other resources.

##### Notes 

Other examples of basic result terms, depending on the test, are reactive, non-reactive and invalid. Extended result messages often explain the meaning and potential limitations of a result.

### Performance

#### 24. Qualitative colorimetric rapid diagnostic tests: agreement of reader with expert visual interpretation

##### Minimal 

≥ 95%.

##### Optimal 

≥ 98%.

##### Notes 

Expert visual interpretation typically requires a panel of skilled operators who directly view the rapid diagnostic test (not a photo of the rapid diagnostic test). For rapid diagnostic tests designed and manufactured for visual interpretation, a reader is unlikely to improve on expert visual interpretation in terms of diagnostic performance metrics such as sensitivity, specificity and limit of detection. A reader’s evaluation should be planned carefully, with consideration of the performance requirements for the assay. Performance with faint lines (low positives) should be assessed, as should the reader’s rates of invalid and indeterminate results as compared with expert visual interpretation.

#### 25. Rapid diagnostic tests other than qualitative colorimetric: performance

##### Minimal and optimal 

Equivalent to state-of-the-art readers.

### Compliance

#### 26. Compliance with medical standards for design and manufacture (medical only)

##### Minimal 

ISO 13 485:2016 Medical devices – Quality management systems – Requirements for regulatory purposes. IEC 61 010–2–101:2018 Safety requirements for electrical equipment for measurement, control and laboratory use – Part 2–101: Particular requirements for in vitro diagnostic medical equipment. Applicable standards associated with the above.

### Data features within the reader

#### 27. Diagnostic data collected

##### Minimal 

Brand and type of test as entered by the user. 

Underlying values and outcome of quality controls (see characteristic 23, that is, help provided by the reader after determination of the result; lay use only). 

Non-medical: Result as entered by the user. 

Result as calculated by the reader. 

Intermediate data used to calculate the result (e.g. intensities of test and control lines).

##### Optimal 

Replace first item in minimal with: brand and type of test, by photographing the test or its packaging. 

Same as rest of minimal, plus: Lot number and expiration date of the test (possibly by photographing the test and its packaging); rapid diagnostic test instruction version; rapid diagnostic test photograph(s) used to calculate the result; specimen type (e.g. whole blood, serum) and volume; and other relevant diagnostic data entered by the user.

##### Notes 

Each health programme should be able to choose whether to require their users to enter lot and expiration data. Because of limited connectivity and storage in many settings, the reader should provide appropriate options for image resolution and cropping. Multiple images may be appropriate: one cropped to the region of the control and test lines for evidence of the result, and another of the entire rapid diagnostic test for supervision of the type of test. Health programmes may prefer to transmit images only in certain cases, such as faint lines and invalid results or during certain research programmes. As noted in characteristic 16, images of the entire rapid diagnostic test may include patient-identifiable information.

#### 28. Patient or case data collected

##### Minimal and optimal 

As determined by the health programme and in compliance with local regulations (e.g. patient identification, location, consent, symptoms).

##### Notes 

Data needs should be balanced with the burden of data entry on users. As noted in the preface, data features can be provided by integrating the reader with a digital health system with those capabilities.

#### 29. Contextual data collected

##### Minimal 

User identification (lay use: if required by the health programme). Location of testing as text entered manually, such as an address or facility name (if enabled by the health programme). Time and date of test. Manufacturer and model name of reader. 

Instrument: Serial number of reader. 

Reader software or firmware version. 

Application: Model of mobile device. Operating system version.

##### Optimal 

Same as minimal plus: Automatic geolocation of test (e.g. via global positioning system, if enabled by the health programme); reader operational data for administration, maintenance and performance metrics (e.g. self-checks, calibration, quality control samples); others as determined by the health programme.

##### Notes 

Almost all these data can and should be collected automatically rather than entered manually.

#### 30. Methods for data entry

##### Minimal 

Typing.

##### Optimal 

Typing. Scanning one-dimensional and two-dimensional barcodes.

##### Notes 

The user can choose one of these methods for entering the types of data listed above. When possible, the reader should instead collect data automatically or enable the user to select from lists, to avoid data-entry errors.

#### 31. Non-volatile memory and storage

##### Minimal 

Professional use: ≥ 200 patient results. ≥ 20 quality control results. 

Lay use: ≥ 50 patient results.

##### Optimal 

Professional use: ≥ 1000 patient results. ≥ 100 quality control results.

##### Notes 

Reader memory is intended as a log of recent results and a temporary repository of results awaiting transmission to a server with the data connectivity features described later. At least certain images should be kept with recent results. See characteristic 29 (that is, contextual data collected) for considerations of image types. Application: This assumes the mobile device has enough space.

#### 32. Role-based access control (professional use only)

##### Minimal and optimal 

Provides access to specific data and reader features for users with different roles.

##### Notes 

Roles may include data managers at several levels (e.g. supervisor, site administrator, national manager) and rapid diagnostic test user (health-care worker).

### Data connectivity

#### 33. Data connectivity methods

##### Minimal 

Instrument: mobile network, Wi-Fi, USB or Bluetooth.

##### Optimal 

Instrument: mobile network and at least one of Wi-Fi, USB, Bluetooth or Ethernet.

##### Minimal and optimal 

Application: mobile network or Wi-Fi as provided by the mobile device.

##### Notes 

Throughout this section, as noted in the preface, data features can be provided by integrating the reader with a digital health system with those capabilities.

#### 34. Handling of intermittent or low-bandwidth connections

##### Minimal 

The user shall be able to perform tests (medical: and receive results) offline, in which case the reader shall transmit those data when back online.

##### Optimal 

Same as minimal, plus: The reader shall transmit automatically (without user action) in the background when back online, prioritizing basic data elements before sending larger, secondary elements such as images.

#### 35. Data exchange standards

##### Minimal 

The reader supports FHIR® or JSON.

##### Optimal 

The reader supports FHIR® and JSON.

##### Notes 

For connections to systems such as laboratory information systems, electronic health records, national registries and surveillance systems.

#### 36. Data destination

##### Minimal and optimal

The health programme shall be able to choose the destination(s) of the reader’s data.

##### Notes 

The reader’s manufacturer and other groups should require permission from the health programme to receive any data.

#### 37. Data ownership

##### Minimal and optimal 

The health programme shall be able to manage the reader in compliance with local regulations on data ownership.

#### 38. Security and privacy

##### Minimal and optimal 

To facilitate use by health programmes in accordance with the laws, regulations and policies in their settings and with best practices, the reader shall provide configurable features so that personal data can be: (a) gathered transparently to users and patients, including consent; (b) collected and processed only for purposes compatible with the health programme’s purposes; (c) limited to what is relevant and necessary; (d) collected accurately; (e) stored in identifiable form no longer than necessary; and (f) secured for integrity and confidentiality, with encryption at rest and in transmission.

##### Notes 

(a)–(f) are adapted from the European Union General Data Protection Regulation (GDPR) 2016/679 (GDPR), article 5, sec. 1. Note that not all the GDPR is relevant or appropriate to this reader in these settings.

### Pricing and accessibility

#### 39. Pricing applicable to all public programmes, nongovernmental organizations and international organizations in low- and middle-income countries

##### Minimal and optimal 

Pricing should be as low as sustainably possible while maintaining quality, based on evidence of the cost of goods sold plus a reasonable profit margin, ensuring security of supply and a fair return on investment for suppliers. Pricing should be affordable, transparently published and inclusive of all fees, including any for warranties, support and software updates.

## Conclusion

With input from invited experts and the public, we have developed a target product profile for automatic readers of rapid diagnostic tests to address a range of possible uses. The product profile is intended to promote the development of effective, useful, sustainable rapid diagnostic test readers in support of health programmes worldwide.
